# Reevaluating Symbiotic Digestion in Cockroaches: Unveiling the Hindgut’s Contribution to Digestion in Wood-Feeding Panesthiinae (Blaberidae)

**DOI:** 10.3390/insects14090768

**Published:** 2023-09-14

**Authors:** Melbert Schwarz, Gaku Tokuda, Haruka Osaki, Aram Mikaelyan

**Affiliations:** 1 Department of Entomology and Plant Pathology, North Carolina State University, 100 Derieux Place, Raleigh, NC 27695, USA; mtschwa2@ncsu.edu (M.S.); hosaki@ncsu.edu (H.O.); 2 Center of Molecular Biosciences, Tropical Biosphere Research Center, University of the Ryukyus, Nishihara-cho, Okinawa 903-0213, Japan; tokuda@comb.u-ryukyu.ac.jp; 3 Department of Agriculture, Kyoto University, Oiwake-cho, Kitashirakawa, Sakyo-ku, Kyoto 606-8502, Japan

**Keywords:** wood-feeding, saproxylophagy, cockroaches, Panesthiinae, Blaberidae, Percoll, lignocellulose, xylanase, cellulase, digestion

## Abstract

**Simple Summary:**

The remarkable ability of insects to digest wood has long fascinated researchers due to its rarity and complexity. Wood-feeding termites and passalid beetles have been extensively studied as paradigms, unveiling intricate symbiotic interactions involving endogenous enzymes and gut microbiomes. However, the wood-digesting capabilities of panesthiine cockroaches remain vastly unexplored, offering a unique opportunity to gain fresh insights into this unique dietary specialization. In this study, we investigated cellulase and xylanase activity in the crop, midgut, and hindgut lumens of *Panesthia angustipennis* and *Salganea taiwanensis*. Utilizing Percoll density gradient centrifugation, we further fractionated the luminal fluid to further elucidate how the activities are partitioned. The crop emerged as a prominent site for fiber digestion, which agrees with previous reports. However, our findings challenge conventional notions, revealing a significant role for the hindgut, which contributes around one-fifth of cellulase and xylanase activity. Particle-associated and potentially bacterial enzymes dominate the hindgut, which is similar to the digestion strategies of certain termites and passalid beetles. Our study sheds light on the remarkable adaptability of wood-feeding insects and provides valuable clues to their evolutionary success on this challenging, nutrient-poor resource.

**Abstract:**

Cockroaches of the subfamily Panesthiinae (family Blaberidae) are among the few major groups of insects feeding on decayed wood. Despite having independently evolved the ability to thrive on this recalcitrant and nitrogen-limited resource, they are among the least studied of all wood-feeding insect groups. In the pursuit of unraveling their unique digestive strategies, we explored cellulase and xylanase activity in the crop, midgut, and hindgut lumens of *Panesthia angustipennis* and *Salganea taiwanensis*. Employing Percoll density gradient centrifugation, we further fractionated luminal fluid to elucidate how the activities in the gut lumen are further partitioned. Our findings challenge conventional wisdom, underscoring the significant contribution of the hindgut, which accounts for approximately one-fifth of cellulase and xylanase activity. Particle-associated enzymes, potentially of bacterial origin, dominate hindgut digestion, akin to symbiotic strategies observed in select termites and passalid beetles. Our study sheds new light on the digestive prowess of panesthiine cockroaches, providing invaluable insights into the evolution of wood-feeding insects and their remarkable adaptability to challenging, nutrient-poor substrates.

## 1. Introduction

Wood is characteristically difficult to utilize and digest as a food source due to its recalcitrance and limited nitrogen content [1]. Despite these formidable nutritional obstacles, certain insect groups, including cockroaches, termites, and beetles, have independently evolved the remarkable ability to harness it as a viable resource [2]. There is remarkable diversity in the mechanisms of digestion among wood-feeding insects [3,4]. Termites are the best studied of all described groups of wood feeders and are characterized by a complex mechanism of symbiotic digestion that includes endogenous components from the insect (including mandibles for the physical breakdown of wood and enzymes produced in the foregut or midgut) combined with enzymes produced by their associated gut microbiome [4]. More recent studies in *Odontotaenius disjunctus* suggest that passalid beetles have also evolved an analogous approach to symbiotic lignocellulose digestion [5,6,7].

Wood-feeding cockroaches of the subfamily Panesthiinae (Blaberidae) evolved the ability to feed on wood independently of termites [8] and present a potentially intriguing exception to the digestive “division of labor” observed in termites and passalid beetles. In a seminal study on *Panesthia cribrata*, Scrivener and colleagues (1989) [9] localized cellulase activity in the anterior midgut, foregut, and gastric caeca but observed almost no activity in the posterior midgut and hindgut regions. The skew in the localization of cellulase activity in the foregut and midgut, combined with the absence of cellulolytic protozoa, was the basis for hypothesizing that the cockroach primarily relies on endogenous enzymes for cellulose digestion. A follow-up study by Scrivener and Slaytor (1994) [10] further indicated a limited contribution of the hindgut to cellulose digestion. However, the methodology used in these studies predominantly assays secreted cell-free enzymes and overlooks contributions from the “hidden [11]”, cell-associated enzymes that are often part of the digestive strategies employed by anaerobic bacteria [12,13]. This inclusion of cell-associated enzymes in the assay of enzyme activity is a fundamental step towards a complete understanding of the hindgut’s role in wood digestion in panesthiine cockroaches.

Studies on the intestinal microbial ecology of *Panesthia angustipennis* and *Salganea esakii* suggested a greater role for the hindgut in the digestion of lignocellulose by panesthiine cockroaches [14,15]. Of the three major gut regions (crop, midgut, and hindgut) investigated, the hindgut of *P. angustipennis* harbors the densest microbiome (31 × 10^9^ cells/g), characterized by a high degree of fermentative activity [14]. Although enzyme activity was not directly assayed in either study, it has been hypothesized that fiber-associated bacteria in the hindgut of panesthiine cockroaches could be playing a role in symbiotic wood digestion [14,15]. If true, this would suggest a degree of convergence in the mechanism of symbiotic digestion in both termites [11,16] and passalid beetles [7]. The lack of clarity about the role of the hindgut in lignocellulose digestion in panesthiine cockroaches warrants a detailed analysis of the distribution of digestive activity, specifically measuring the contribution of both soluble and cell-associated enzymes.

In this study, we present a comprehensive examination of enzyme activity associated with the breakdown of cellulose and xylan in different gut compartments of two species of panesthiine cockroaches, *Panesthia angustipennis* and *Salganea taiwanensis*. By investigating cellulase and xylanase activity in the crop, midgut, and hindgut, we aim to shed light on the potential contribution of hindgut bacteria and refine our understanding of wood digestion in panesthiine cockroaches.

## 2. Materials and Methods

### 2.1. Insects

Adult specimens (of both sexes) of *P. angustipennis* and *S. taiwanensis* were collected from decaying hardwood on Ishigaki Island, Japan. The insects were housed in plastic containers containing the same rotting wood they were collected with and maintained at a temperature of 23 °C. Prior to dissection, the cockroaches were immobilized by placing them on ice to facilitate the subsequent dissection process. Sterilized dissection tools soaked in 70% ethanol were used to carefully dissect the insects, separating the pleura to expose the thoracic and abdominal cavities. The intact gut was then meticulously removed and further divided into three distinct compartments, namely the crop, midgut, and hindgut. Luminal fluid from each gut compartment was collected by longitudinally cutting the gut compartment and preserving it in 1× phosphate-buffered saline (PBS). Luminal fluid, collected from each replicated gut region, was separated into three parts. The first part was used to measure the total enzyme activity in the lumen of the respective compartment. The second part underwent fractionation using Percoll density gradient centrifugation (see Section 2.2). Additionally, the third part was reserved for DNA extraction for a separate study. The experiment was conducted in triplicate. Each replicate for *P. angustipennis* consisted of two individuals, while each replicate for *S. taiwanensis* consisted of three individuals.

### 2.2. Enrichment and Separation of Wood Fibers from Gut Compartments

To enrich the wood fibers present in the luminal fluid of each gut compartment, we employed a previously established method [16]. Briefly, a stock solution of 1× Percoll (Cytiva, Uppsala, Sweden) was prepared in sterile 1× phosphate buffered saline (PBS) and subsequently filter-sterilized using a 0.22 μm syringe filter. This stock solution was further diluted to obtain a 90% Percoll working solution. In a sterile 2.0-milliter microcentrifuge tube, we carefully added 1.8 mL of the 90% Percoll solution to form a continuous density column. Subsequently, 100 μL of pooled luminal contents from two *P. angustipennis* or three *S. taiwanensis* specimens were gently layered over the continuous Percoll gradient and subjected to centrifugation (Himac CF 16RX, Hitachi Koki, Tokyo, Japan) at 20,000× *g* at 4 °C for 30 min. This centrifugation process resulted in the formation of two distinct bands within the Percoll column (Figure S1 provides a schematic representation of the separation of luminal fluid using the Percoll column). The upper opaque layer composed of cells and the lower, brown-colored layer containing wood particles were referred to as the “fiber-free” and “fiber” fractions, respectively. Using a micropipette, these fractions were carefully transferred to fresh tubes. The ~200-microliter fractions were rinsed with 1.6 mL of sterile 1× PBS and centrifuged at 20,000× *g* and 4 °C for 30 min. After discarding the supernatant, the rinsing step was repeated twice. Finally, the rinsed fractions were resuspended in 200 μL of sterile 1× PBS to obtain the enriched fractions for downstream analysis.

### 2.3. Preparation of Crude Enzyme Extracts

In order to systematically assay enzymatic contributions from all potential pools of xylanase and cellulase activity in the gut lumen, our approach was to prepare crude enzyme extracts that included (1) cell-free or soluble enzymes released by sonication and (2) cell-wall or membrane-associated enzymes released by detergent extraction. All crude extracts were prepared for enzyme assays as described in Mikaelyan et al. (2014) [16]. Briefly, for both luminal fluid and wash fractions, 200 μL of each sample was first sonicated using an ultrasonic homogenizer (VP-5S sonicator, Titech, Saitama, Japan) at 25% intensity, using six cycles of 5 s with a 10-s pause between cycles. Any resultant debris was pelleted by centrifugation at 20,000× *g* for 10 min at 4 °C. A ~200-microliter aliquot of the supernatant was collected as the sonication extract in a sterile tube and stored at 4 °C until use. The pellet was washed three times with 100 μL protease inhibitor solution (Complete Mini, EDTA-free, Roche, Mannheim, Germany), then resuspended in 100 μL CelLyticB (Sigma-Aldrich, St. Louis, MO, USA) by vortexing it for 15 s to ensure the complete extraction of any potentially cell wall or membrane-associated enzymes. The final suspension was then placed on ice for 10 min and briefly centrifuged. The supernatants from this step were collected in a fresh microcentrifuge tube.

### 2.4. Quantification and Analysis of Enzymatic Activity

Assays of cellulase and xylanase activity in the luminal fluid and Percoll fractions followed the protocol outlined in Schwarz et al. (2023) [7]. The pH of the enzyme reactions was buffered using 0.1 M HEPES buffers (Dojindo, Kumamoto, Japan) and adjusted to reflect the conditions in the respective gut compartments: the crop (pH 5), midgut (pH 6), and hindgut (pH 7) of *P. angustipennis* (based on values determined by Bauer et al., 2015 [14]). These buffers were used to prepare the cellulose and xylan substrates for the enzyme assays.

For the assessment of cellulase and xylanase activities, 30 μL of the sonicated or detergent extracts from each sample were subjected to incubation with 200 μL of 2.0% [w:v] microcrystalline cellulose (Sigmacell Cellulose Type 20, Sigma) or 0.025% xylan from beechwood (Sigma), respectively. The reaction mixtures were gently agitated for a duration of one hour at 37 °C, allowing for enzymatic activity to occur. Following the incubation period, the release of reducing sugar equivalents was quantitatively determined at 660 nm on a GeneQuant 1300 spectrophotometer (GE Healthcare, Buckinghamshire, UK), following the methodology outlined by Jue and Lipke (1985) [17] with modifications based on Mikaelyan et al. (2014) [16]. The enzyme activities measured in the two (fiber + fiber-free) Percoll fractions collectively constitute what is denoted as “particle-associated activity” subsequently in this study.

To establish quantitative measurements, standard curves were generated using glucose (for cellulase) or xylose (for xylanase) as references, as described by Schwarz et al. (2023) [7]. These standard curves allowed for the determination of the reducing sugar content in the reaction mixtures. One unit of enzyme activity was defined as the quantity of enzyme required to release 1 μmol of reducing sugar equivalents from the respective substrate (cellulose or xylan) per minute per gram of insect.

Relevant pairwise comparisons were performed to assess the differences between groups using the Kruskal-Wallis test. The analysis was conducted using R software (version 4.0.4; R Core Team, 2021) [18] with the ‘kruskal.test’ function from the ‘stats’ package. The test compares the medians of the groups and provides a test statistic (H), degrees of freedom (df), and a *p*-value. A significance level of 0.05 was used to determine statistical significance. If the *p*-value was less than 0.05, it indicated a significant difference between the groups.

## 3. Results and Discussion

**Figure 1:**
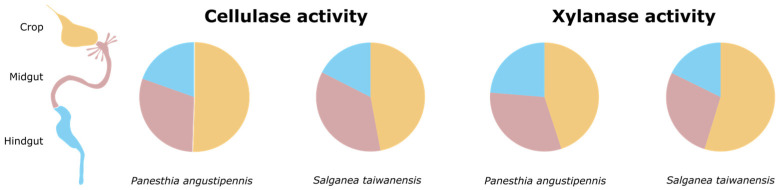
Proportion of cellulase and xylanase activity in the lumen of the gut regions of *Panesthia angustipennis* and *Salganea taiwanensis*. The legend is a color-coded generalized schematic of the panesthiine gut, indicating the regions sampled for the enzyme assays.

**Figure 2:**
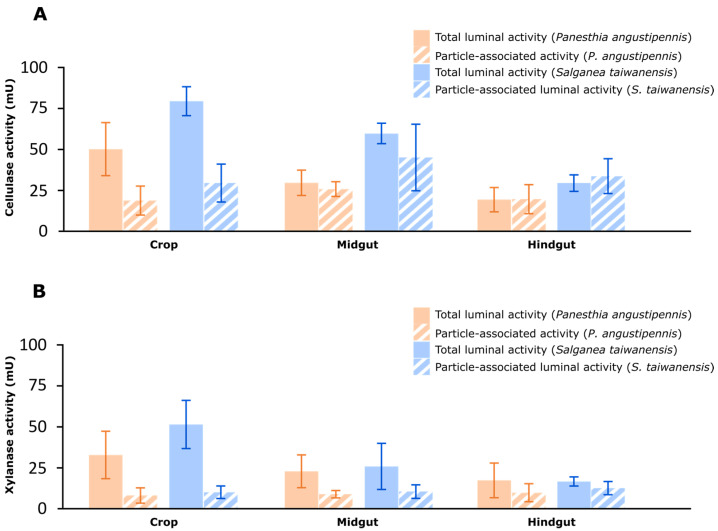
The distribution of cellulase (**A**) and xylanase (**B**) activity in the lumen of the crop, midgut, and hindgut of *Panesthia angustipennis* and *Salganea taiwanensis*. One unit of enzyme activity is defined as 1 μmol of sugar equivalent released per minute, per gram of insect. Particle-associated activity refers to the sum of cellulase or xylanase activity associated with the fiber and fiber-free fractions obtained from density gradient centrifugation of the luminal fluid.

Our study provides compelling evidence for the significant roles of the luminal fluids played by all three gut regions in the digestion of cellulose and xylan in *Panesthia angustipennis* and *Salganea taiwanensis*. While the luminal fluid in the crop contributes the most to this process, housing cellulase activities of 79.97 ± 8.86 mU (*S. taiwanensis*) and 50.60 ± 16.26 mU (*P. angustipennis*) and xylanase activities of 51.47 ± 14.72 mU (*S. taiwanensis*) and 32.83 ± 14.50 mU (*P. angustipennis*) (Figure 1; Tables S1 and S2), the midgut plays a lesser yet notable role. Specifically, our results reveal that the luminal fluid in the midgut of *S. taiwanensis* houses approximately 60.21 ± 6.26 mU of cellulase activity and 10.44 mU of xylanase activity (Figure 2A,B; Tables S1 and S2). In comparison, the midgut of *P. angustipennis* contains around 29.95 ± 7.77 mU of cellulase activity and 22.86 ± 10.02 mU of xylanase activity. Furthermore, the luminal fluid in the hindgut of *S. taiwanensis* houses approximately 29.76 ± 5.07 mU of cellulase activity and 16.65 ± 2.79 mU of xylanase activity, while that in the hindgut of *P. angustipennis* houses about 19.61 ± 7.45 mU of cellulase activity and 17.30 ± 10.58 mU of xylanase activity.

Our results (Figure 1) support the notion put forth by Scrivener et al. (1989) [9] and Scrivener and Slaytor (1994) [10] that the foregut is the major contributor to lignocellulose digestion, contributing as much as 65.1% in *P. cribrata*. The luminal fluid in the crop of *S. taiwanensis* retained as much as 47.1% of the total luminal cellulase activity and 54.8% of the total luminal xylanase activity assayed across all three gut regions (Figure 1; Tables S1 and S2). The crop of *S. taiwanensis* was found to have significantly higher luminal cellulase (H = 3.8571, df = 1, *p*-value = 0.04953) activity compared to that of *P. angustipennis*; however, no significant difference was observed for xylanase activities (H = 2.3333, df = 1, *p*-value = 0.1266). Regardless, *P. angustipennis* showed a similar pattern in the distribution of the proportion of luminal cellulase and xylanase activities as *S. taiwanensis*, with the crop retaining 50.5% and 45%, respectively (Figure 1). Since the crop is positioned at the beginning of the digestive tract, it might be strategically advantageous to expose ingested wood fibers to a high density of cellulase and xylanase activities, especially given that the particles likely do not spend a substantial amount of time in the foregut [19]. We can also speculate that the enzymes in the crop help with the pre-treatment of lignocellulose to make its sequential digestion in the midgut and hindgut easier. A related phenomenon has been observed in the industrial treatment of cellulosic material, where synergistic xylanase and endoglucanase activity helped “open up” cellulose fibers, thereby improving access to cellulose [20]. Regardless, this skew in the distribution of cellulase and xylanase activity towards the crop in both species suggests that this is indeed characteristic of the subfamily Panesthiinae but appears to be also typical of all lignocellulose-feeding Blaberidae, such as the leaf-feeding *Geoscapheus dilatatus* (Geoscapheinae; formerly Panesthiinae) and *Calolampra elegans* (Epilamprinae) [21]. Since cellulase and xylanase activities in the luminal fluid decreased toward the hindgut in both *P. angustipennis* and *S. taiwanensis*, there is likely no supplementary addition of cell-free digestive enzymes in the midgut or hindgut.

Our use of Percoll fractionation to separately assay soluble and particle-associated enzyme activity allows us to further theorize about the potential contributors to symbiotic wood digestion. As a result, particle-associated cellulase activity accounted for 37.7% and 37.3% of the total cellulase activity in the crop of *P. angustipennis* and *S. taiwanensis*, respectively (Figure 2; Table S1), which suggests that most of the activity in the crop is cell-free or extracellular in nature. Similar results for xylanase activity suggest that hemicellulose digestion in the crop is also predominantly conducted by cell-free enzymes (Figure 2; Table S2). Although disentangling the specific contributions of the insect’s salivary glands versus those of its microbial symbionts to this cell-free enzyme activity calls for further investigation, to the best of our knowledge, the genomes of Blattodea, including termites, sequenced to date do not encode typical xylanase genes.

Contributing about a third of the total cellulase and xylanase activity, the midgut in both cockroach species is a notable contributor to the breakdown of wood. The *S. taiwanensis* midgut was associated with significantly higher (H = 3.8571, df = 1, *p*-value = 0.04953) cellulase activity than *P. angustipennis*. However, both species had statistically comparable xylanase activities (H = 0.047619, df = 1, *p*-value = 0.8273). Specifically, the midgut of *P. angustipennis* retained 29.9% of the total cellulase activity and 31.3% of the total xylanase activity (Figure 1; Tables S1 and S2). Similarly, the retention of 35.4% and 27.5% of cellulase and xylanase activity, respectively, in the midgut of *S. taiwanensis* suggests a comparable significance of the midgut to lignocellulose digestion (Figure 1). These findings align with those from *P. cribrata*, where the midgut was observed to house about 33.4% of cellulase activity [9]. Percoll fractionation of the cellulase activity in the midgut lumen shows that, unlike the situation presented in the crop, a majority (87.2% and 75.5%, respectively, in *P. angustipennis* and *S. taiwanensis*) of the activity in both insects is particle-associated (Figure 2; Table S1). Given that the midgut of *P. angustipennis* hosts a dense [14], actively fermenting microbiome (11.9 × 10^9^ cells/g), it seems plausible that this activity is bacterial in origin. Xylanase activity, however, does not appear to be distributed the same way as cellulase activity, since only half as much (38.8% and 40.4%, respectively, in *P. angustipennis* and *S. taiwanensis*) of the total xylanolytic activity appears to be particle-associated (Figure 2; Tables S1 and S2).

Our analysis of the digestive contributions of the hindgut exhibited the most notable deviation from the previous narrative; contrary to Scrivener et al. (1989) [9], who attributed only 1.1% of the total cellulase activity in *P. cribrata* to the hindgut, our results suggest that the hindgut of *P. angustipennis* and *S. taiwanensis* retains 19.6% and 17.5%, respectively (Figure 1; Table S1). Both species had statistically similar levels of cellulase (H = 2.3333, df = 1, *p*-value = 0.1266) and xylanase (H = 0.047619, df = 1, *p*-value = 0.8273) activities. In both species, particle-associated activities accounted for more than half of the total cellulase activities in the hindgut (Figure 2; Table S1). It strongly suggests a dominant role for the hindgut microbiome in the digestion of cellulose and xylan.

The observed disparity between our results from *P. angustipennis* and *S. taiwanensis* and previous studies in *P. cribrata* [9,10] can be mainly attributed to the inclusion of a detergent extraction step in our preparation of crude enzyme extracts, which facilitated the extraction of cell-associated enzymes (Tables S3–S6). This extraction method appears to have a more significant impact on the differences observed compared to the use of sonication, as reported by Mikaelyan et al. (2014) [16]. In their study on *N. corniger*, they found that while only 11% and 33% of the total cellulase activity in the hindgut was released by homogenization and sonication, respectively, the majority of the activity was accessible only through detergent extraction. Likewise, in the anterior hindgut of the passalid beetle *Odontotaenius disjunctus*, detergent extraction revealed 46% and 61% of total cellulase and xylanase activity, respectively [7]. Furthermore, variations in buffer pH used for enzyme assays may also contribute to the observed differences between our results and previous studies [9,10]. Unlike previous studies using a consistent 0.1 M acetate buffer at pH 5.5, we adjusted the pH of our enzyme buffers to match the conditions in the respective gut compartments of *P. angustipennis*, recognizing the potential impact of pH on enzymatic activity in different gut regions [11].

Although the proportion of particle-associated fraction of xylanase activity appears to be lower than that of cellulase activity (Figure 2; Tables S1 and S2), this difference was not statistically significant for either species (*P. angustipennis,* H = 2.3333, df = 1, *p*-value = 0.1266; *S. taiwanensis,* H = 1.1905, df = 1, *p*-value = 0.2752). In addition, it is noteworthy that particle-associated xylanase activity was similar in all gut compartments, regardless of cockroach species. Therefore, the common mechanism of bacterial xylan degradation may be present in panesthiine cockroaches.

The predominance of particle-associated cellulase and xylanase activities in both *P. angustipennis* and *S. taiwanensis* points to the presence of symbiotic wood digestion mechanisms analogous to what has been observed in distantly related wood-feeding insect groups such as wood-feeding higher termites [11,16,22] and passalid beetles [7]. In both termites [16,22] and passalid beetles [7], substantial cellulase and xylanase activities associated with bacterial cells in the hindgut have been documented. As in the hindgut of termites, a previous study indicated that the center of all gut compartments in *P. angustipennis* is anoxic [14]. It is therefore plausible to speculate that the pool of putatively cell-associated enzyme activity observed in *P. angustipennis* and *S. taiwanensis* is likely contributed by multienzyme complexes, such as cellulosomes [12] or similar multiprotein complexes found in bacteria involved in the anaerobic breakdown of plant material [23]. This notion finds further support in previous reports of high abundances of Clostridia and Bacteroidota in the hindgut as well as the midgut of *P. angustipennis* and *Salganea esakii* that have been implicated in fiber digestion [14,15,24]. Future efforts to characterize the microbiomes associated with wood particles in panesthiine cockroaches should help identify those bacterial taxa that are most closely associated with fiber digestion. Given the importance of fiber-associated bacteria to digestion in herbivorous mammals [25] and the broad similarity in microbiome composition between cockroaches and mammals [24,26], an understanding of the contribution of microbes to wood digestion in the Panesthiinae can contribute to a better understanding of symbiotic lignocellulose digestion in ruminants and other herbivores.

The heightened cell-free xylanase activities observed in the crop of panesthiine cockroaches, in contrast to other gut compartments, pose a captivating conundrum, particularly in light of the potential absence of endogenous xylanase genes within the Blattodea order. Two hypotheses may be postulated to explain this observation. The first entails the potential involvement of fungal-derived xylanases, given that these cockroaches have a predilection for highly decomposed wood [10,27]. A potential role for fungi in the predigestion of wood has also been recently suggested for passalid beetles that colonize wood in similar states of decomposition [7]. Despite a prior study dismissing the role of fungal cellulases in cellulose digestion in the gut of *P. cribrata* [10], the enigmatic contribution of fungal xylanases remains an open and intriguing question. The second hypothesis explores the possibility of gene duplication events within cellulase genes, which may have led to a shift in substrate specificity towards xylan, akin to the evolutionary trajectory observed in stick insects [28]. Considering the presence of multiple cellulase gene homologs within members of Blattodea [29] and the resemblance in chemical structure between cellulose and xylan, it is conceivable that some have acquired xylanolytic functionality in panesthiine cockroaches.

Although gut residence times for panesthiine cockroaches were not measured in our study, it is worth noting that food particles in the gut of similarly sized and distantly related blaberids, *Periplaneta americana,* reportedly pass at different rates in different regions of the gut [19]. After passing rapidly through the foregut (spending less than 30 min) and the midgut (where they spend around 1.5 h), they proceed to be slowly fermented in the hindgut for over 18 h [19]. The residence time of plant fibers is a critical component in the efficient anaerobic degradation of lignocellulose in intestinal environments [1,30]. Presumably, the steep variations in residence times between different gut regions are critical to the effective contribution of each compartment to the overall digestion of wood fibers in cockroaches. Detailed investigations could also provide valuable context for the dynamics of the contributions of the host and the microbiome to the overall digestive process in the Panesthiinae.

Our investigation into cellulase and xylanase activities in panesthiine cockroaches (*S. taiwanensis* and *P. angustipennis*) provides valuable insights into their wood-digesting capabilities and allows for comparisons with other well-studied wood-feeding insects. Compared to the values obtained from the midguts and hindguts of termites within the genus *Nasutitermes*, which produce around 50–200 mU of cellulase [11,16] and almost 1800 mU of xylanase [22], it becomes evident that panesthiine cockroaches have slightly lower activities (especially for xylanase). Cellulase and xylanase activities we observed in the midguts of panesthiine cockroaches are more comparable to those of other well-studied wood-feeding insects. For instance, the midgut of the wood-feeding passalid beetle *O. disjunctus* houses 14.35 mU of cellulase and 73.15 mU of xylanase activity, while its hindgut houses 24.45 mU of cellulase activity and 173.5 mU of xylanase activity [7], indicating a higher concentration of these enzymes in the hindgut compared to panesthiine cockroaches. This suggests that panesthiine cockroaches have similar enzymatic activities in their midguts as the passalid beetle, despite the differences in hindgut cellulase activity between the two groups. Overall, these comparisons shed light on the distinct enzymatic capabilities of different wood-feeding insects, highlighting the diverse strategies they employ to digest lignocellulosic materials. At the same time, the repeated evolution of broadly similar strategies, such as the reliance on fiber-associated communities in termites [16], passalid beetles [7], and likely panesthiine cockroaches, suggests that the potential solutions to the nutritional challenges of wood digestion in insects are also somewhat limited in number. Further research into the complexities of symbiotic lignocellulose digestion in blaberid cockroaches, especially close non-wood-feeding relatives of the Panesthiinae, will help us better understand the adaptations that enable these insects to thrive on fiber-rich diets.

## Data Availability

Data will be made available upon request.

## Supplementary Materials

The following supporting information can be downloaded at: https://www.mdpi.com/article/10.3390/insects14090768/s1. Figure S1. A schematic representation of the Percoll column used to fractionate the luminal fluid from different gut compartments into a fiber-free fraction (enriched in microbial cells) and a fiber fraction (enriched in wood particles and their associated microbial cells); Table S1. Cellulase activities in different gut regions of *Panesthia angustipennis* and *Salganea taiwanensis*, including particle-associated activity and proportion in the lumen. Enzyme activities measured both in the lumen and the particle-associated portion of each gut region are presented in milliunits (mU). The particle-associated activity represents the combined activities of sonication and detergent extracts from each sample (for a detailed split of the particle-associated cellulase activity, please refer to Tables S3 and S5). One unit of enzyme activity is defined as 1 μmol of sugar equivalent released per minute, per gram of insect. The percentage of enzyme activity in the lumen that is particle-associated is also presented. To enhance the visualization of the cellulase activity patterns across the different gut regions and species, the cells in the table have been conditionally formatted as a heatmap, with 0 mU represented by white, the 50th percentile by black, and 80 mU represented by green; Table S2. Xylanase activities in different gut regions of *Panesthia angustipennis* and *Salganea taiwanensis*, including particle-associated activity and proportion in the lumen. Enzyme activities measured both in the lumen and the particle-associated portion of each gut region are presented in milliunits (mU). The particle-associated activity represents the combined activities of sonication and detergent extracts from each sample (for a detailed split of the particle-associated xylanase activity, please refer to Tables S4 and S6). One unit of enzyme activity is defined as 1 μmol of sugar equivalent released per minute, per gram of insect. The percentage of enzyme activity in the lumen that is particle-associated is also presented. To enhance the visualization of the cellulase activity patterns across the different gut regions and species, the cells in the table have been conditionally formatted as a heatmap, with 0 mU represented by white, the 50th percentile by black, and 80 mU represented by green; Table S3. Cellulase activities measured in crude extracts from various sources in *Panesthia angustipennis*. The sources include sonication, detergent treatment of luminal fluids, and Percoll fractions (fiber and fiber-free) obtained from different gut regions (crop, midgut, and hindgut). Enzyme activity is quantified as 1 μmol of sugar equivalent released per minute, per gram of insect. The combined activities of sonication and detergent extracts from each sample are referred to as ‘particle-associated activity’ in the manuscript; Table S4. Xylanase activities measured in crude extracts from various sources in *Panesthia angustipennis*. The sources include sonication, detergent treatment of luminal fluids, and Percoll fractions (fiber and fiber-free) obtained from different gut regions (crop, midgut, and hindgut). Enzyme activity is quantified as 1 μmol of sugar equivalent released per minute, per gram of insect. The combined activities of sonication and detergent extracts from each sample are referred to as ‘particle-associated activity’ in the manuscript; Table S5. Cellulase activities measured in crude extracts from various sources of *Salganea taiwanensis*. The sources include sonication, detergent treatment of luminal fluids, and Percoll fractions (fiber and fiber-free) obtained from different gut regions (crop, midgut, and hindgut). Enzyme activity is quantified as 1 μmol of sugar equivalent released per minute, per gram of insect. The combined activities of sonication and detergent extracts from each sample are referred to as ‘particle-associated activity’ in the manuscript; Table S6. Xylanase activities measured in crude extracts from various sources in *Salganea taiwanensis*. The sources include sonication, detergent treatment of luminal fluids, and Percoll fractions (fiber and fiber-free) obtained from different gut regions (crop, midgut, and hindgut). Enzyme activity is quantified as 1 μmol of sugar equivalent released per minute, per gram of insect. The combined activities of sonication and detergent extracts from each sample are referred to as ‘particle-associated activity’ in the manuscript.
