# Supplementary material for: Reevaluating Symbiotic Digestion in Cockroaches: Unveiling the Hindgut’s Contribution to Digestion in Wood-Feeding Panesthiinae (Blaberidae)
Source: Insects. 2023 Sep 14;14(9):768. doi: 10.3390/insects14090768 (PMC10531843; doi:10.3390/insects14090768)
Supplement: Supplementary file 1 [file insects-14-00768-s001.zip › Figure_S1.pdf]

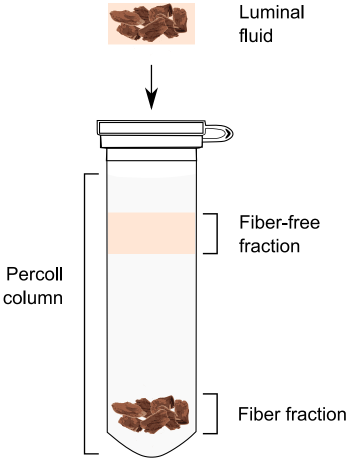

Figure S1. A schematic representation of the Percoll column used to fractionate the luminal fluid from different gut compartments into a fiber-free fraction (enriched in microbial cells) and the fiber fraction (enriched in wood particles and their associated microbial cells).
